# Uso de la evaluación externa de la calidad en las fases extraanalíticas de los laboratorios clínicos españoles: una encuesta de la Sociedad Española de Medicina de Laboratorio (SEQC^ML^)

**DOI:** 10.1515/almed-2024-0160

**Published:** 2025-04-03

**Authors:** Andrea Caballero Garralda, Immaculada Comas Reixach, Carlos García Miralles, Rubén Gómez Rioja, María Antonia Llopis Díaz, Débora Martínez Espartosa, Reyes Nicolás de Blas, Mariona Panadès Turró, Laura Puigví Fernández, Laura Rodelgo Jiménez, Berta Sufrate-Vergara, Emma Ventura Orriols

**Affiliations:** Extraanalytical Quality Commission of the Spanish Society of Laboratory Medicine (SEQC^ML^), Department of Clinical Biochemistry, Vall d’Hebron Hospital, Barcelona, España; Extraanalytical Quality Commission of the Spanish Society of Laboratory Medicine (SEQC^ML^), Department of Clinical Biochemistry, Parc Tauli Hospital Universitari, Sabadell, España; Extraanalytical Quality Commission of the Spanish Society of Laboratory Medicine (SEQC^ML^), Department of Laboratory Medicine, La Paz-Cantoblanco-Carlos III Hospital, Madrid, España; Extraanalytical Quality Commission of the Spanish Society of Laboratory Medicine (SEQC^ML^), Catalonian Institute of Health, Barcelona, España; Extraanalytical Quality Commission of the Spanish Society of Laboratory Medicine (SEQC^ML^), Department of Clinical Biochemistry, Clínica Universidad de Navarra, Madrid, España; Extraanalytical Quality Commission of the Spanish Society of Laboratory Medicine (SEQC^ML^), Department of Clinical Biochemistry, Ramón y Cajal University Hospital, Madrid, España; Extraanalytical Quality Commission of the Spanish Society of Laboratory Medicine (SEQC^ML^), Quality manager-Spanish Society of Laboratory Medicine (SEQC^ML^), Barcelona, España; Extraanalytical Quality Commission of the Spanish Society of Laboratory Medicine (SEQC^ML^), Extraanalytical Head Department, Director of Operations, CLILAB Diagnòstics, Vilafranca del Penedès, Barcelona, España; Extraanalytical Quality Commission of the Spanish Society of Laboratory Medicine (SEQC^ML^), Institut of Laboratory medicine, San Carlos Clinic Hospital, Madrid, España; Extraanalytical Quality Commission of the Spanish Society of Laboratory Medicine (SEQC^ML^), Extraanalytical Department of Laboratory, Hospital Sant Joan de Deu, Esplugues de Llobregat, Barcelona, España

**Keywords:** fase extraanalítica, indicadores de calidad, programa de evaluación externa de la calidad, encuesta

## Abstract

**Objetivos:**

La gestión de indicadores de calidad (IC) es una herramienta óptima para monitorizar y mejorar el desempeño en las fases extraanalíticas del Laboratorio Clínico (LC). Por este motivo, la Sociedad Española de Medicina de Laboratorio (SEQC^ML^) lanzó en 2001 el Programa de Preanalítica.

**Métodos:**

En 2023 se realizó una encuesta para evaluar el grado de participación en el Programa y valorar la necesidad de mejora, así como conocer el procedimiento de la recogida y cálculo de IC extraanalíticos.

**Resultados:**

El 51 % de los encuestados (124 respuestas en total) participaba en el Programa, y el motivo más importante para hacerlo era porque se considera necesario monitorizar las fases extraanalíticas (38 %). Por el contrario, la principal razón para no participar fue la dificultad para recoger los datos (32 %). Respecto a las necesidades de mejora del Programa, el 56 % considera útil la revisión de indicadores disgregada por tipo de extracción (ambulatoria vs. urgente) y el 86 % tendría interés en participar en un programa de postanalítica. En cuanto a otros indicadores interesantes para ser incluidos, la mayoría se relacionaban con incidencias en los tiempos y temperaturas de transporte desde centros de extracción periféricos.

**Conclusiones:**

Existe un creciente interés en los IC relacionados con las fases pre y postanalíticas, sin embargo, un número reducido de laboratorios obtienen datos completos con regularidad. Los hallazgos de esta encuesta han permitido guiar las futuras ampliaciones del Programa de Preanalítica para adaptarse a las necesidades actuales de los LC.

## Introducción

La mayoría de los errores del laboratorio clínico (LC) ocurren en las fases pre-y postanalíticas del proceso total de un test (PTT), lo que se traduce en un riesgo elevado para la seguridad del paciente [1]. Aunque el concepto del *brain-to-brain loop* fue descrito hace más de 43 años [2], la concienciación y el consenso sobre el impacto de los aspectos extraanalíticos del LC en los resultados se ha alcanzado recientemente [3], 4]. Todos los pasos del PTT deben ser monitorizados y evaluados de manera continua para asegurar que los resultados del LC tienen la calidad suficiente para ser informados [5].

El establecer un sistema de monitorización de calidad interno [6] y la participación en programas externos de aseguramiento de la calidad (EQA), son herramientas esenciales para la mejora continua [7]. La gestión de indicadores de calidad (IC), como parte de la estrategia de mejora continua, se ha demostrado que es una herramienta óptima para vigilar y mejorar el desempeño en las fases extraanalíticas [8], 9]. De hecho, de acuerdo con la norma ISO 15189:2023, la identificación y uso de IC en todas las fases del PTT es un requisito esencial para la acreditación [10], idealmente revisando todo el proceso, incluyendo los procedimientos pre-y postanalíticos [11].

Por este motivo, la Sociedad Española de Medicina de Laboratorio (SEQC^ML^) lanzó en 2001 el Programa de Preanalítica, inicialmente en forma de estudios de corte transversal [12], 13], pasando a partir de 2014 a un Programa de intercomparación de IC preanalíticos. El Programa se centra en un número reducido de IC basado en las incidencias más prevalentes detectadas en la fase previa del Programa, con especial atención sobre los motivos de rechazo de las muestras que se producen dentro del laboratorio, y solo se tienen en cuenta los resultados de rutina, no las muestras urgentes. Además, el denominador utilizado es el tipo de prueba mayoritaria en el tipo de espécimen (creatinina para suero, hemograma para EDTA y tiempo de protrombina para citrato, solo para orina se usa número total de tubos). Se trata de IC muy orientados al proceso de toma (venopunción) y recogida de muestra, donde el principal mecanismo de mejora es la formación del personal implicado en base a una correcta monitorización de los procesos.

El objetivo del Programa es contribuir a la mejora de los procesos preanalíticos, disminuyendo errores que puedan tener impacto en la seguridad del paciente. La mayoría de los IC se han mantenido estables durante los años, lo que ha permitido generar especificaciones basadas en el estado del arte, de acuerdo con las recomendaciones de la *European Federation of Laboratory Medicine* (EFLM) (*Task Finish Group – Performance specifications for the extraanalytical phases*). Según esta recomendación, se adoptan los percentiles p25, p50 y p75 de los resultados de los participantes como especificaciones óptimas, deseables y mínimas, respectivamente.

Por otro lado, debido a que cada vez más los LC se centran en cómo conseguir que los resultados de laboratorio tengan el mayor impacto posible en los resultados de los pacientes, se está poniendo un mayor foco en la fase postanalítica, y cómo el laboratorio puede añadir valor a los resultados (comentarios interpretativos, aviso valores críticos, tiempos de respuesta, etc.) [14], 15]. En este sentido, el *Working Group for Postanalytics of the Croatian Society of Medical Biochemistry and Laboratory Medicine* llevó a cabo una encuesta para comprobar el manejo de la fase postanalítica y las recomendaciones nacionales [16].

Además, las nuevas tecnologías, los sistemas de información de laboratorio (SIL) y la mayor automatización ponen de manifiesto la necesidad de revisar si los indicadores actuales son válidos para monitorizar el desempeño del laboratorio actual. A este respecto el *Working Group for the Preanalytical Phase* de la EFLM llevó a cabo una encuesta sobre cómo los laboratorios europeos monitorizaban la fase preanalítica [17], 18], encontrando que el 71 % de los laboratorios participaban en algún EQA de extraanalítica. Sin embargo, en el ámbito de los laboratorios participantes en los diferentes esquemas del programa de garantía de calidad de la SEQC^ML^ (PGCLC), la participación en el programa de extraanalitica es mucho más baja.

Por este motivo, se llevó a cabo una encuesta entre los LC participantes en algún esquema del PGCLC. El objetivo de la encuesta fue evaluar el grado de participación en el Programa y valorar la necesidad de mejorar la calidad del mismo, así como conocer el procedimiento y las dificultades a las que los laboratorios hacen frente en relación a la recogida y cálculo de IC extraanalíticos y, por último, si los IC actuales satisfacían las necesidades de los LC actuales, con mayor grado de automatización, complejidad e informatización.

## Materiales y métodos

### Diseño de la encuesta

La encuesta fue diseñada por los miembros de la Comisión de Calidad Extraanalítica de la SEQC^ML^, incluía 48 preguntas en total, tanto con respuesta si/no, como respuesta múltiple y respuesta abierta, y fue creada con la plataforma *Google forms*. La encuesta se difundió a los miembros de la comisión como prueba piloto y después se redefinió con las sugerencias aportadas. Todas las preguntas y las respuestas posibles se muestran en el Material Suplementario, Tabla 1.

**Tabla 1:** Resumen de los principales resultados de la encuesta.

Peticiones anuales	
**Respuestas (** **n=124)**	**%**
<25.000	17
25.000-300.000	41
>300.000	42

**Tipos de peticiones**	

**Respuestas (** **n=124)**	**%**
Ambulatoria de rutina	31
Ambulatoria de urgencias	17
Hospitalarias de rutina	26
Hospitalarias de urgencias	26

**Porcentaje de muestras externas**	

**Respuestas (** **n=124)**	**%**
<33 %	26
33–66 %	43
>66 %	31

**Revisión de indicadores desagregada respecto al tipo de extracción**	

**Respuestas (** **n=124)**	**%**
Sí	56
No	44

**Interés en incluir sigma**	

**Respuestas (n=120** ^**a**^ **)**	**%**
Sí	74
No	26

**Interés programa postanalítica**	

**Respuestas (** **n=124)**	**%**
Sí	86
No	14

**Incluir laboratorios de urgencias**	

**Respuestas** ^**b**^	**%**
Sí, combinando urgencias y rutina	37
Sí, separando urgencias y rutina	44
No	19

^a^De los laboratorios que participan en otros programas, 1 sí tiene sigma y 3 no. ^b^Las 4 respuestas restantes son laboratorios que participan en otros programas y todos indican que sí evalúan muestras urgentes.

### Difusión de la encuesta

La encuesta se difundió por correo electrónico a los laboratorios participantes en el PGCLC (n=2.409) y también a través de un post en LinkedIn, en ambos casos se indicaba el link para responder la encuesta a través de *Google forms*. El cuestionario se dejó abierto durante dos meses y medio, desde el 19 de junio de 2023 al 31 de agosto de 2023.

### Análisis de la encuesta

Todos los resultados de preguntas cerradas fueron analizados a través de gráficos, las respuestas abiertas se analizaron una a una y agruparon según si compartían un tema en común.

## Resultados

Se obtuvieron 124 respuestas, lo que supone una tasa de respuesta del 5 %, similar a la de otras encuestas realizadas previamente por la comisión [19]. Los resultados más destacados se resumen en la Tabla 1.

En cuanto a las características de los encuestados, el 42 % pertenecían a laboratorios de tamaño grande con más de 300,000 peticiones anuales, 41 % a laboratorios medianos (25.000–300.000) y el resto de laboratorios eran pequeños, de menos de 25.000 peticiones/año. El reparto de tipo de peticiones era equitativo entre ambulatorias de rutina, hospitalarias de rutina y ambulatorias y hospitalarias de urgencias. En cuanto al porcentaje de muestras recibidas de centros externos (extracción fuera de las instalaciones donde se encuentra el laboratorio), la mayoría (43 %) recibía entre un 33–66 % de muestras externas.

**Figura 1:**
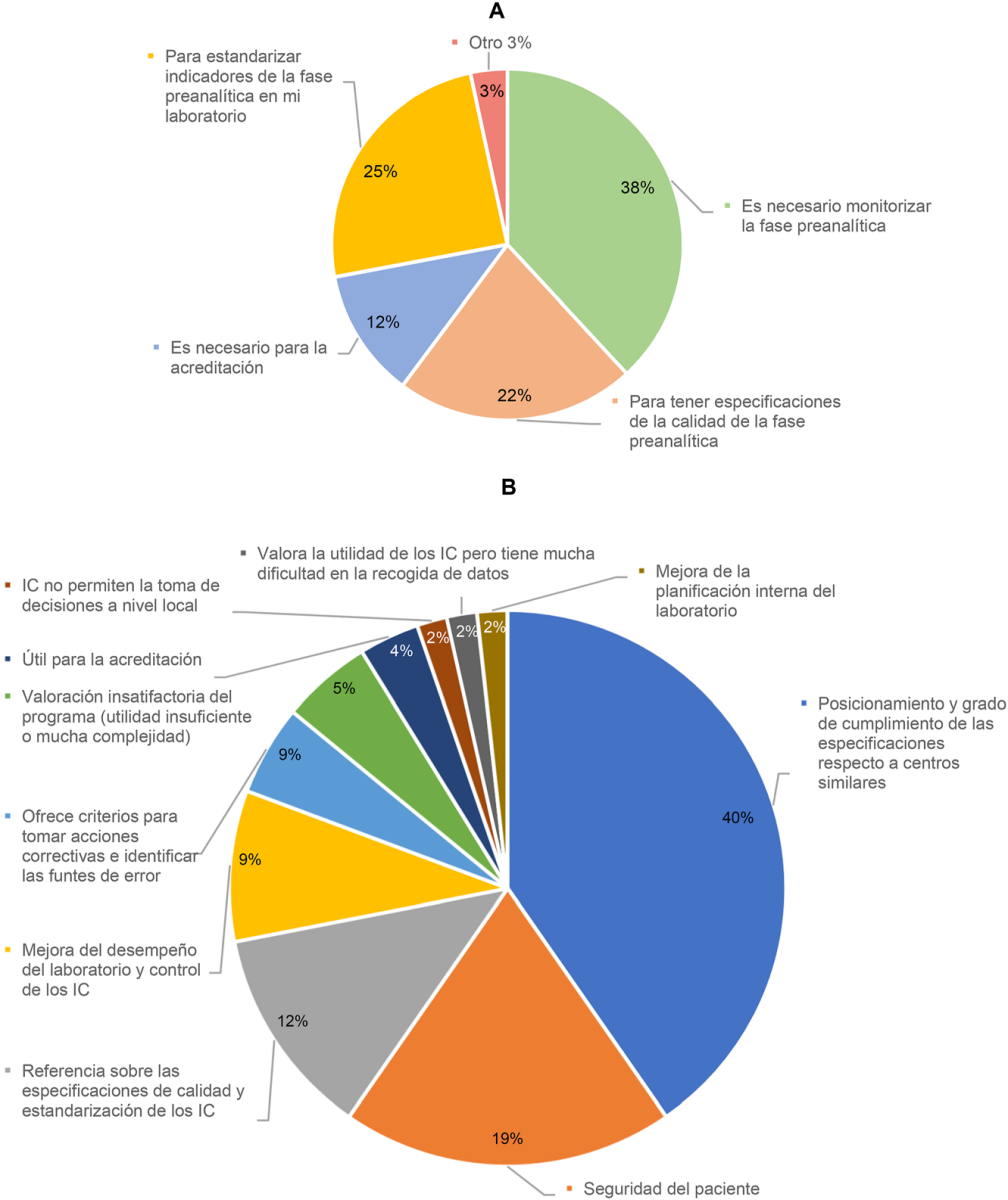
Resultados de los laboratorios que participan en el Programa de Preanalítica (63 laboratorios). (A) Motivos para participar. (B) Principales aportaciones del programa.

**Figura 2:**
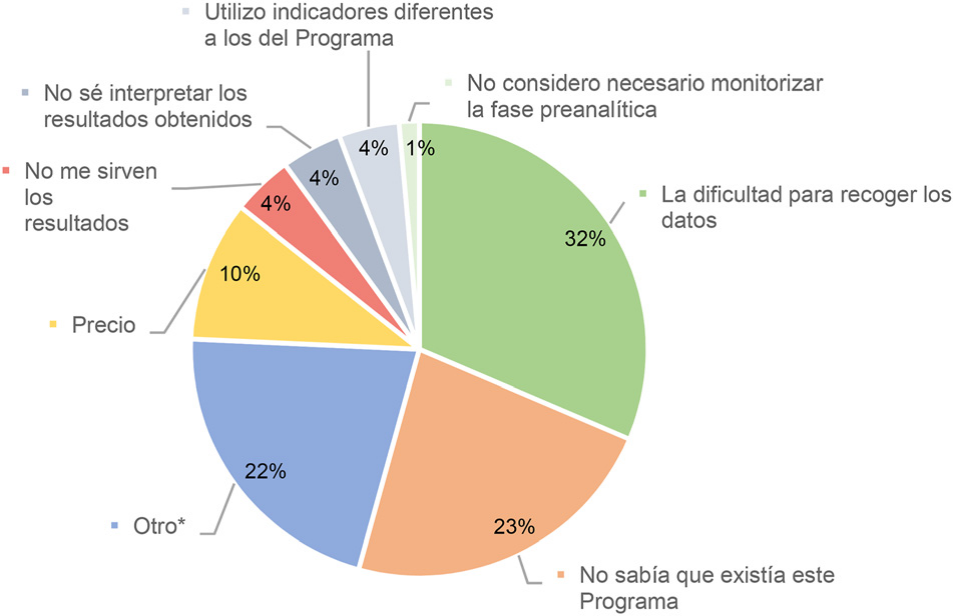
Motivos para no participar de los laboratorios que no participan en el Programa de Preanalítica (57 laboratorios). *Otro: (1) No tener los índices reflejados en el SIL. (2) No participa la empresa a la que pertenece el Laboratorio. (3) Actualmente nos gustaría que nuestros clientes participaran. (4) Ninguno en especial. (5) Participé hace tiempo y no estaba muy de acuerdo con los Informes de resultados. (6) Solo estamos inscritos en el Programa de sangre oculta en heces. (7) Trabajo en el laboratorio de urgencias. (8) Falta de tiempo. (9) Monitorizamos fase preanalítica pero solo a nivel interno. (10) No tenemos propuestas de organizadores. (11) Las especificaciones de calidad utilizadas. (12) No disponemos de la determinación de índices séricos en el laboratorio y además no tenemos informatizado el sistema de petición de volantes ni emisión de informes.

De todos los participantes en la encuesta, el 51 % participa en el Programa de Preanalítica, frente al 46 % que no participa en ningún programa de Extraanalítica y un 3 % que participa en programas de otra organización. Entre los participantes, el principal motivo para hacerlo es porque se considera necesario monitorizar la fase extraanalítica (38 %), seguido de estandarizar IC (25 %) y tener especificaciones de la calidad (22 %). El motivo menos seleccionado fue la necesidad para el proceso de acreditación. De entre los participantes, el 40 % considera que la aportación fundamental del Programa es proporcionar posicionamiento y grado de cumplimiento de las especificaciones respecto a centros similares (40 %) y seguridad del paciente (19 %) (Figura 1). Por el contrario, el principal motivo para no participar en el Programa, de los 57 laboratorios que en la encuesta afirmaron no hacerlo, fue la dificultad para recoger los datos (32 %), seguido del desconocimiento de la existencia del Programa (23 %) (Figura 2).

**Figura 3:**
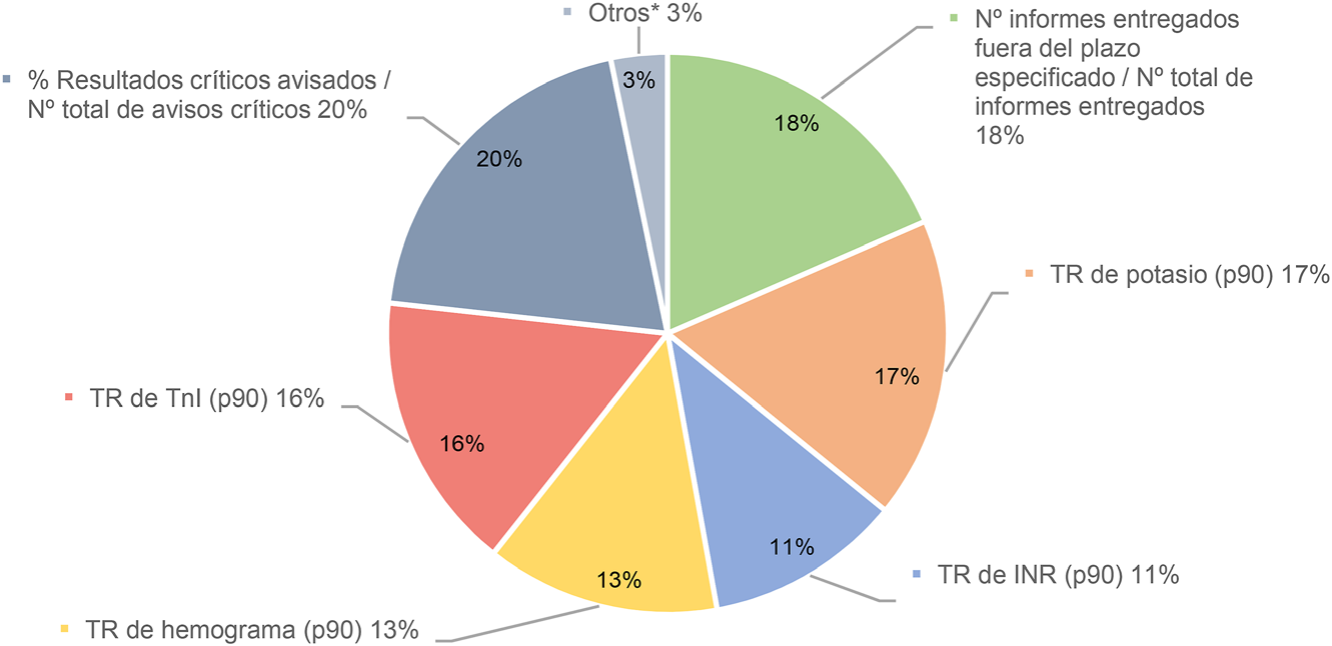
Interés en indicadores postanalíticos. TR: tiempo de respuesta desde la recepción en el laboratorio hasta la emisión de resultados en muestras urgentes. *Otros: (1) Indicadores relacionados con consultas de clínicos al laboratorio y con avisos de resultados, (2) Hospitalizados y ambulatorios de rutina, (3) Porcentaje de informes modificados/ número total de informes, (4) Rechazos críticos avisados oportunamente/ Número total de rechazos críticos detectados, (5) Número de informes mal emitidos-validados/ número total informes, (6) Tiempo de respuesta desde la recepción en el laboratorio hasta la emisión del resultado de gasometrías venosos y arteriales, (7) Indicadores relacionados con la emisión del resultado de la procalcitonina en muestras urgentes, (8) Resultados de interpretación/opinión diagnóstica (% de falta de correlación entre resultados vinculantes y % de correlación entre el cuadro clínico y los análisis realizados), (9) Indicadores relacionados con la transcripción de resultados (% de transcripción errónea de resultados y % de transcripción inoportuna de resultados).

Respecto a las necesidades de mejora del programa, el 56 % afirmó que sí le sería útil la revisión de indicadores disgregada respecto al tipo de extracción (ambulatoria vs. hospitalización). Más en concreto, en cuanto a la inclusión de las peticiones de urgencias en el cálculo de IC, la mayoría de los laboratorios estarían interesados en incluirlas y de mantener separada a las de la rutina, el 37 % las combinaría y el 19 % no las incluiría. En todos los Programas diferentes a los de la SEQC^ML^ se contabilizan las muestras urgentes. El 74 % vería interesante incluir el cálculo del sigma, de los laboratorios que participan en otros programas (4 en total), solo uno de ellos cuenta con el cálculo del sigma. Destaca que el 86 % de los encuestados tendría interés en participar en un Programa de Postanalítica, y se preguntó sobre el interés de incluir diferentes indicadores relacionados con esta fase, en una pregunta abierta sobre qué otros indicadores incluirían (Figura 3). Los indicadores de elección fueron los relacionados con el porcentaje de avisos críticos realizado respecto al total de valores críticos avisables y el número de informes entregados fuera del tiempo de respuesta. Otros indicadores que los participantes sugirieron fueron los relacionados con la modificación de informes, comentarios interpretativos, transcripción de resultados y emisión de informes con errores.

La segunda parte de la encuesta se centró en el proceso de recogida y cálculo de IC por parte de los laboratorios. En el 73 % de los casos se realiza a través del SIL, seguido de un contaje manual (hoja de cálculo Excel o similar) en un 19 % de los casos y el resto a través de softwares específicos. En cuanto a proveedores de SIL, los mayoritarios son Modulab^®^ de Werfen (23 %), Infinity^®^ de Roche (19 %) y Servolab^®^ de Siemens Healthineers (14 %), el resto presentaban porcentajes inferior al 10 %.

Por último, en relación a otros indicadores no incluidos en el Programa y que serían interesantes para los encuestados, las respuestas fueron diversas pero la mayoría se relacionaban con incidencias en los tiempos y temperaturas de transporte desde centros de extracción periféricos o con indicadores ya incluidos en el Programa, pero aplicados a otros tipos de muestras (jeringas de gasometrías, orinas para urocultivo, otras muestras de microbiología, etc.). Respecto a los aspectos a mejorar, las respuestas vuelven a reflejar el interés por indicadores relacionados con el transporte, la mayoría de los laboratorios reciben entre un 33 % y 66 % de muestras externas, lo que implica una necesidad considerable de coordinación con centros externos, así como incluir datos del laboratorio de urgencias, mejorar la explicación de cómo se deben recoger los indicadores, cambiar el denominador de cálculo de los indicadores e incluir otro tipo de muestras.

## Discusión

La participación en Programas de Garantía Externa de la Calidad de la fase preanalítica es una herramienta útil para identificar y priorizar las áreas de mejora. El Programa de evaluación de la calidad de la fase preanalítica de la SEQC^ML^ es una herramienta fundamental, y así lo reflejan los participantes con sus respuestas, la mayoría de ellos siendo grandes laboratorios, con más de 300.000 peticiones/día.

Para gestionar adecuadamente los errores en la fase preanalítica es indispensable monitorizarlos a través de unos buenos IC, además es necesario poder compararse con el resto de laboratorios para disponer de especificaciones que permitan evaluar el desempeño, con el objetivo final de disminuir los errores y garantizar la seguridad del paciente.

De los resultados se deduce que la participación de los LC españoles en Programas de Extraanalítica, en general, sigue siendo baja. Comparando con una participación del 71 % en una encuesta europea similar (17), solo la mitad de los laboratorios participan en España. El motivo principal declarado para no participar es la dificultad para la recogida de los datos, seguido de cerca del desconocimiento de la existencia del Programa. Otra posibilidad que se contempla para explicar la baja participación en los Programas de Extraanalítica es la reticencia a exponer datos internos de la organización, que puedan afectar negativamente a la imagen o a la relación con los clientes. A pesar de que el programa asegura la confidencialidad, puede existir un sesgo negativo a la no participación por este motivo.

Por un lado, a partir de este estudio, se pusieron en marcha campañas en la web del PGCLC y en las principales redes sociales de la SEQC^ML^. Por otro lado, los resultados de la encuesta sacan a relucir lo relevante y esencial que resulta la colaboración con los principales proveedores de SIL para crear módulos para el cálculo de indicadores que sean automatizados, estandarizados, a tiempo real y sencillos de compartir y evaluar. Como se ha observado, el 19 % de los laboratorios depende de métodos manuales para el cálculo de IC, lo cual supone una oportunidad para implementar soluciones más automatizadas a través del SIL.

En cuanto al interés de los participantes en incluir las muestras de urgencias e indicadores de postanalítica y transporte de muestras, desde la Comisión de Calidad Extraanalítica y el Comité de Programas Externos de la Calidad de la SEQC^ML^ se realizó una prueba piloto entre los miembros, después de la cual, se ha decidido ampliar al resto de participantes en el Programa a lo largo del 2025, indicadores con muestras urgentes. Para ello se han duplicado los indicadores actuales y los participantes obtendrán dos informes, uno como el actual (muestras de rutina) y otro para muestras de urgencias. Así los LC participantes podrán comparar ambos tipos de procesos y establecer medidas correctivas más enfocadas y dirigidas.

En una segunda fase, en base a las respuestas y al interés general actual en la fase postanalítica y otros indicadores de preanalítica diferentes a los actuales, se pretende incluir dos IC más: cumplimiento del tiempo de respuesta y aviso de resultados críticos, y, por último, se añadirá otro indicador preanalítico: incidencias en el transporte desde centros de extracción periféricos, siguiendo el mismo esquema de prueba piloto entre los participantes e implementación progresiva en 2026.

Respecto a otros programas, el *Working Group Laboratory Errors and Patient Safety* (WG-LEPS) de la *International Federation of Clinical Chemistry* (IFCC) no ha hecho ninguna nueva publicación desde el 2019 [20] y el programa *Australian key incident monitoring and management system program* (KIMMS) del *Royal College of Australasian Pathologist* (RCPA) [21] desde 2020, los resultados de ambos ya se compararon con el programa de Preanalítica de la SEQC^ML^ en una publicación de 2022 [22]. No obstante, existen dos experiencias recientes a comentar, una de la Fundación de Bioquímica Argentina [23] y otra en China [24]. En cuanto a la primera, se trata de un subprograma dentro de otro, es una encuesta cuatrimestral, con pacientes ambulantes y solo cuatro indicadores, la forma de recolectar los indicadores es en un archivo Excel. La respuesta es voluntaria, con lo que el porcentaje de laboratorios participantes varía y, además, depende del indicador. Se muestran los resultados entre 2021–2022: entre 100–200 de un total de 400 laboratorios responden (25–50 %) y comparando sus percentiles con los de la IFCC, los resultados son parecidos (23). En cuanto al Programa organizado por el *National Centre for Clinical Laboratories of China* se realizó el envío vía email durante el 2017–2019 de dos cuestionarios al año, evaluando seis indicadores, 434 laboratorios de la provincia de Zhejiang respondieron a los seis ciclos. La tasa de rechazo es muy baja en comparación con el programa Q-Probes y Q-Tracks del *College of American Pathologist* (CAP).

Una limitación importante de la encuesta presentada en este artículo es la ya mencionada baja participación. Aunque no es algo nuevo de este estudio y no se puede conocer la causa exacta (¿sobreexposición a encuestas?, ¿tiempo limitado de los facultativos de laboratorio?, ¿bajo interés por el tema?, etc.), sí merece la pena ser comentado y valorar un cambio de formato para futuros sondeos, la realización de una mayor difusión y el acceso más sencillo a través de diferentes canales de comunicación, para así mejorar la tasa de respuestas y la potencia de los estudios.

Existe un creciente interés en los IC relacionados con las fases pre y postanalíticas, sin embargo, queda reflejado en la encuesta que un reducido número de LC obtienen datos completos con regularidad. No obstante, los hallazgos de esta encuesta han permitido guiar los futuros desarrollos y optimizaciones del Programa de Preanalítica de la SEQC^ML^ para adaptarse a las necesidades actuales de los Laboratorios Clínicos. La dificultad en la recogida de los datos es el principal motivo de la baja participación, por lo que es necesaria la colaboración con proveedores de SIL para el desarrollo de nuevas herramientas que permitan una recogida de datos sencilla. Si bien, es responsabilidad de los laboratorios destinar más recursos, de personas y económicos, a la fase extraanalítica.

En conclusión, para gestionar adecuadamente los errores en la fase preanalítica es indispensable monitorizarlos a través de unos buenos IC, además es necesario poder compararse con el resto de laboratorios para disponer de especificaciones que permitan evaluar el desempeño, con el objetivo final de disminuir los errores y garantizar la seguridad del paciente.

## Supplementary Material

Supplementary Material

## Material Suplementario

Este artículo incluye material suplementario (https://doi.org/10.1515/almed-2024-0160).
