# Supplementary material for: Uso de la evaluación externa de la calidad en las fases extraanalíticas de los laboratorios clínicos españoles: una encuesta de la Sociedad Española de Medicina de Laboratorio (SEQCML)
Source: Adv Lab Med. 2025 Apr 3;6(2):206–12. [Article in Spanish] doi: 10.1515/almed-2024-0160 (PMC12107416; doi:10.1515/almed-2024-0160)
Supplement: Supplementary file 1 — Supplementary Material [file j_almed-2024-0160_suppl_001.docx]

**MATERIAL SUPLEMENTARIO**

**Tabla 1.** Preguntas encuesta**.**

**¿Actualmente participa en el Programa de Garantía Externa de la Calidad de Preanalítica de la SEQC-ML?**

1. Sí
2. No, participo en un Programa de Garantía Externa de la Calidad de la fase preanalítica de otra organización
3. No, no participo en ningún Programa de Garantía Externa de la Calidad de la fase preanalítica

**Sí que participa en el Programa de Preanalítica de la SEQC-ML**

**¿Cuál es el principal motivo por el que participa?**

1. Es necesario monitorizar la fase preanalítica
2. Para tener especificaciones de la calidad de la fase preanalítica
3. Es necesario para la acreditación
4. Para estandarizar indicadores de la fase preanalítica en mi laboratorio
5. Otro

**En el caso de que el laboratorio recoja indicadores de la fase preanalítica diferentes a los del Programa, indique cuales son:**

**¿Cómo recoge los datos necesarios para el cálculo de los indicadores?**

1. A través del Sistema Informático del Laboratorio
2. A través de un contaje manual en Excel (o similar)
3. Solicitando los datos a Sistemas de Información del centro
4. Otro

**¿Cuál es el proveedor de su SIL?**

**Cuando recibe los resultados, ¿su laboratorio revisa los indicadores de forma desagregada respecto al tipo de centro de extracción? Por ejemplo, ¿si tiene extracciones en diferentes centros de Atención primaria y un indicador sale elevado, revisa el resultado del indicador en cada uno de los centros?**

1. Sí
2. No

**¿Qué le aporta el Programa de Preanalítica?**

**¿Qué aspectos de interés añadiría o modificaría?**

**¿Le sería útil que se incluyera el cálculo de Sigma en el Programa?**

1. Sí
2. No

**¿Le sería útil si el Programa se ampliara para muestras urgentes?**

1. Sí, sería útil añadir los indicadores para muestras urgentes junto a las de rutina en el mismo Programa
2. Sí, sería útil un Programa de Preanalítica de muestras urgentes independiente del actual de muestras de rutina
3. No

**¿Estaría interesado en participar en un Programa de Postanalítica?**

1. Sí
2. No

**¿Qué indicadores de la fase postanalítica le serían más útiles?**

1. Número de informes entregados fuera del plazo especificado / Número total de informes entregados
2. Tiempo de respuesta desde la recepción en el laboratorio hasta la emisión del resultado de potasio en muestras urgentes (p90)
3. Tiempo de respuesta desde la recepción en el laboratorio hasta la emisión del resultado de INR en muestras urgentes (p90)
4. Tiempo de respuesta desde la recepción en el laboratorio hasta la emisión del resultado de hemograma en muestras urgentes (p90)
5. Tiempo de respuesta desde la recepción en el laboratorio hasta la emisión del resultado de TnI en muestras urgentes (p90)
6. Porcentaje de resultados críticos avisados / Número total de avisos críticos
7. Otro

**Respecto a su laboratorio, marque el número de peticiones que reciben anualmente**

1. < 25.000
2. 25.000 - 300.000
3. 300.000

**Respecto a su laboratorio, seleccione el tipo de peticiones que reciben**

1. Ambulatorias de rutina
2. Ambulatorias de urgencias
3. Hospitalarias de rutina
4. Hospitalarias de urgencias

**Respecto a su laboratorio, ¿qué porcentaje de muestras externas reciben? Se entiende como externa aquella muestra no extraída en el centro.**

1. < 33%
2. 33 - 66%
3. 66%

**Nombre de su laboratorio**

**Comentarios:**

**Participa en un Programa de Garantía Externa de la Calidad de la fase preanalítica con otra organización**

**¿En qué Programa participa?**

**¿Qué aspectos le aporta su Programa respecto al Programa de Preanalítica de la SEQC-ML?**

**¿Qué aspectos de interés añadiría o modificaría de su Programa?**

**¿Cuál es el principal motivo para participar en un Programa de la fase preanalítica?**

1. Es necesario monitorizar la fase preanalítica
2. Para tener especificaciones de la calidad de la fase preanalítica
3. Es necesario para la acreditación
4. Para estandarizar indicadores de la fase preanalítica en mi laboratorio
5. Otro

**En el caso de que el laboratorio recoja indicadores de la fase preanalítica diferentes a los del Programa, indique cuales son:**

**¿Cómo recoge los datos necesarios para el cálculo de los indicadores?**

1. A través del Sistema Informático del Laboratorio
2. A través de un contaje manual en Excel (o similar)
3. Solicitando los datos a Sistemas de Información del centro
4. Otro

**¿Cuál es el proveedor de su SIL?**

**Cuando recibe los resultados, ¿su laboratorio revisa los indicadores de forma desagregada respecto al tipo de centro de extracción? Por ejemplo, ¿si tiene extracciones en diferentes centros de Atención primaria y un indicador sale** elevado, revisa el resultado del indicador en cada uno de los centros?

1. Sí
2. No

**¿Su Programa incluye el cálculo de Sigma?**

1. Sí
2. No

**¿Su Programa evalúa muestras urgentes?**

1. Sí
2. No

**¿Estaría interesado en participar en un Programa de Postanalítica?**

1. Sí
2. No

**¿Qué indicadores de la fase postanalítica le serían más útiles?**

1. Número de informes entregados fuera del plazo especificado / Número total de informes entregados
2. Tiempo de respuesta desde la recepción en el laboratorio hasta la emisión del resultado de potasio en muestras urgentes (p90)
3. Tiempo de respuesta desde la recepción en el laboratorio hasta la emisión del resultado de INR en muestras urgentes (p90)
4. Tiempo de respuesta desde la recepción en el laboratorio hasta la emisión del resultado de hemograma en muestras urgentes (p90)
5. Tiempo de respuesta desde la recepción en el laboratorio hasta la emisión del resultado de TnI en muestras urgentes (p90)
6. Porcentaje de resultados críticos avisados / Número total de avisos críticos
7. Otro

**Respecto a su laboratorio, marque el número de peticiones que reciben anualmente**

1. < 25.000
2. 25.000 - 300.000
3. 300.000

**Respecto a su laboratorio, seleccione el tipo de peticiones que reciben**

1. Ambulatorias de rutina
2. Ambulatorias de urgencias
3. Hospitalarias de rutina
4. Hospitalarias de urgencias

**Respecto a su laboratorio, ¿qué porcentaje de muestras externas reciben? Se entiende como externa aquella muestra no extraída en el centro.**

1. < 33%
2. 33 - 66%
3. 66%

**Nombre de su laboratorio**

**Comentarios:**

**No participa en ningún Programa de Garantía Externa de la Calidad de la fase preanalítica**

**¿Cuál es el motivo para no participar en Programas de Garantía Externa de la Calidad de la fase preanalítica?**

1. Precio
2. No considero necesario monitorizar la fase preanalítica
3. No me sirven los resultados
4. No sé interpretar los resultados obtenidos
5. La dificultad para recoger los datos
6. No sabía que existía este Programa
7. Utilizo indicadores diferentes a los del Programa
8. Otro

**En el caso de que el laboratorio recoja indicadores de la fase preanalítica diferentes a los del Programa, indique cuales son:**

**¿Cómo recoge los datos necesarios para el cálculo de los indicadores?**

1. A través del Sistema Informático del Laboratorio
2. A través de un contaje manual en Excel (o similar)
3. Solicitando los datos a Sistemas de Información del centro
4. Otro

**¿Cuál es el proveedor de su SIL?**

**Cuando recibe los resultados, ¿su laboratorio revisa los indicadores de forma desagregada respecto al tipo de centro de extracción? Por ejemplo, ¿si tiene extracciones en diferentes centros de Atención primaria y un indicador sale elevado, revisa el resultado del indicador en cada uno de los centros?**

1. Sí
2. No

**¿Le sería útil que se incluyera el cálculo de Sigma en el Programa?**

1. Sí
2. No

**¿Le sería útil si el Programa se ampliara para muestras urgentes?**

1. Sí, sería útil añadir los indicadores para muestras urgentes junto a las de rutina en el mismo Programa
2. Sí, sería útil un Programa de Preanalítica de muestras urgentes independiente del actual de muestras de rutina
3. No

**¿Estaría interesado en participar en un Programa de Postanalítica?**

1. Sí
2. No

**¿Qué indicadores de la fase postanalítica le serían más útiles?**

1. Número de informes entregados fuera del plazo especificado / Número total de informes entregados
2. Tiempo de respuesta desde la recepción en el laboratorio hasta la emisión del resultado de potasio en muestras urgentes (p90)
3. Tiempo de respuesta desde la recepción en el laboratorio hasta la emisión del resultado de INR en muestras urgentes (p90)
4. Tiempo de respuesta desde la recepción en el laboratorio hasta la emisión del resultado de hemograma en muestras urgentes (p90)
5. Tiempo de respuesta desde la recepción en el laboratorio hasta la emisión del resultado de TnI en muestras urgentes (p90)
6. Porcentaje de resultados críticos avisados / Número total de avisos críticos
7. Otro

**Respecto a su laboratorio, marque el número de peticiones que reciben anualmente**

1. < 25.000
2. 25.000 - 300.000
3. 300.000

**Respecto a su laboratorio, seleccione el tipo de peticiones que reciben**

1. Ambulatorias de rutina
2. Ambulatorias de urgencias
3. Hospitalarias de rutina
4. Hospitalarias de urgencias

**Respecto a su laboratorio, ¿qué porcentaje de muestras externas reciben? Se entiende como externa aquella muestra no extraída en el centro.**

1. < 33%
2. 33 - 66%
3. 66%

**Nombre de su laboratorio**

**Comentarios:**
